# Editorial: The Human Gutome: Nutrigenomics of Host-Microbiome Interactions

**DOI:** 10.3389/fgene.2016.00158

**Published:** 2016-09-06

**Authors:** Dimiter Dimitrov, Ines Thiele, Lynnette R. Ferguson

**Affiliations:** ^1^Diavita Ltd. VarnaBulgaria; ^2^Luxembourg Centre for Systems Biomedicine, University of LuxembourgEsch-sur-Alzette, Luxembourg; ^3^Discipline of Nutrition and Dietetics, Faculty of Medical and Health Sciences, The University of AucklandAuckland, New Zealand

**Keywords:** precision nutrition, systems medicine, translational medicine, gut microbiome, metabolism, intestinal permeability, mucosal viscosity, dietary phytochemicals

The concept of the “Human Gutome,” as a unifying term for multilevel system interaction between the microbiome and the human digestive tract, was established in 2011 (Dimitrov, 2011). In the effort to overcome low translatability of bench-to-bedside research, two major approaches gained recent attention, namely the computational modeling of human metabolism (Aurich and Thiele, 2016) and organ-on-chip, including gut-on-chip tools (Shah et al., 2016). Human digestion starts in the mouth and the correlation between oral microbial species and health is well established (Lockhart et al., 2012). Previously, we also suggested a practical model of cross-talk among diet, oral microbial pathogens and disease susceptibility (Dimitrov). Precision nutrition takes into account nutritional epidemiology across different ethnic groups, differences in macro and micro nutrient intake during lifespan, snapshot or longitudinal multi-omics profiles with or without nutritional challenges in different phenotypes, as well as personalized tailor-made and/or algorithm-based diets targeting individual nutritional requirements for disease prevention or in conjunction with dedicated dietary recommendations with or without pharmacotherapy (Ferguson et al., 2016). The papers in this issue present inter-disciplinary approaches of research of system biology (Shoaie and Nielsen) and medical practice (Salazar et al.). They look at the dynamic systems of the human body as part of an integrated whole (Magnúsdóttir et al.). Models on extensive experimental data are applied (Adamberg et al.; Ahmed Nasef et al.), and aim to understand disease mechanism and suggest personalized treatments (Tailford et al.).

The “Cross Talks” concept gained recent popularity especially among metabolic diseases researchers. In particular, stepwise, the concept evolved from adipose—muscle to gut-brain-adipose-muscle (Argilés et al., 2005; Bartness et al., 2005; Cegla et al., 2010). Quite the same principle, at present, can be postulated for the human microbiome and gut physiology. Tellez et al. present an excellent example for gathering of several medical subjects: intestinal permeability—mucosal viscosity—gut microbiome. Special value of the cited research is the addition of bone mineral density—a topic that is not often mentioned within the booming microbiome science.

Mathematical modeling as a powerful tool for the organization and analysis of microbiome biological data is reviewed in detail by Shoaie and Nielsen. The current challenge to interrelate different levels of information, from the genome to the transcriptome, the proteome and the metabolome, and experimental data from widely used high-throughput techniques is described as a practical didactical guide of how to recreate a given phenotype, and ultimately to make predictions about network and cellular behavior. A real-life modeling example can be visualized from the paper of Magnúsdóttir et al., where readers from different disciplines, from bioinformaticians to clinicians, are provided with hands-on practical tutorial of computational predictions of how the microbiota contribute to the B-vitamin pool of the gut, and how the human metabolism can benefit from the B-vitamin biosynthesis of the microbiota.

The myriad of clinical changes, including a basal pro-inflammatory state (inflamm-aging), that directly interfaces with the microbiota of older adults and enhances susceptibility to disease accompanying aging is reviewed by Salazar et al. Studies in older adults demonstrate that the gut microbiota correlates with diet and basal level of inflammation. Links exist between the microbiota and a variety of clinical problems plaguing older adults, including physical frailty, *Clostridium difficile* colitis and atherosclerotic metabolic syndrome. Manipulation of the microbiota and microbiome of older adults holds promise as an innovative strategy to influence the development of comorbidities associated with aging.

The starch utilization system from B. thetaiotaomicron has been studied in detail and the way the composition of amino acids affects fructan utilization by *B. thetaiotaomicron* and alters the profile of produced SCFAs in a defined medium is described by Adamberg et al. Their results show that fructan metabolism of *B. thetaiotaomicron* DSM 2079 depends on the amino acid supply. B. thetaiotaomicron is one of the most versatile glycan utilizers in the intestine. Even though the majority of intestinal micro-organisms prefer glycans over proteins as growth substrates, proteins are also utilized, in particular in the distal colon where the availability of carbohydrates is limited because they have been used up in the proximal part of the intestinal tract. In this paper the amino acids metabolized most actively by *B. thetaiotaomicron* were Asp, Ser, and Thr.

Strategies used by mucin-degrading bacteria to utilize host glycans are reviewed by Tailford et al., where readers can navigate through the 20 genes encoding mucins, how those genes express peripheral terminal epitopes for glycan diversity, how different bacterial strains interact via different enzymes to influence cyalic acid, fucose and blood group metabolism. Special attention is paid to the role of mucin-degrading bacteria in modulating the gut inflammatory response at obese and diabetic states. The bacterium *Akkermansia muciniphila* is more common in healthy-weight humans than individuals with obesity and type 2 diabetes. A recent study evaluated the association between facal *A. muciniphila* abundance, facal microbiome gene richness, diet, host characteristics, and their changes after calorie restriction (CR) (Dao et al., 2016). While CR is not always easy to achieve, several small-molecule targets have emerged in the impetuous search for calorie restriction mimetics, of which resveratrol, metformin, and rapamycin are the most extensively studied. Interaction of dietary phytochemicals as resveratrol through the pattern recognition receptors (TRLs, NLRs) with intestinal microbiota are thoroughly described by Ferguson et al. (2016). Bioavailability of dietary phytochemicals is of critical importance if a pharmaceutical approach is applied, and the determination is not only complicated by the biochemical properties of the molecule but also by microbial-mediated metabolism. Humans absorb resveratrol well, but since it is quickly metabolized and eliminated, its' bioavailability is low. Recent findings showed that resveratrol reduces levels of trimethylamine-N-oxide (TMAO), known to be a contributory factor in the development of atherosclerosis (Chen et al., 2016). This was partially mediated through down-regulating the enterohepatic farnesoid X receptor-fibroblast growth factor (FXR) axis, and indicates that gut microbiota may become an interesting target for pharmacological and nutritional precision medicine interventions to decrease the risk of developing metabolic diseases.

In summary, The Human Gutome concept gives researchers, nutritionists, and clinicians a common platform for interdisciplinary data sharing in the evolving microbiome science.

## Author contributions

All authors listed, have made substantial, direct and intellectual contribution to the work, and approved it for publication.

### Conflict of interest statement

The authors declare that the research was conducted in the absence of any commercial or financial relationships that could be construed as a potential conflict of interest.
